# Interleukin 1β receptor blocker (Anakinra) and regenerative stem cell therapy: two novel approaches effectively ameliorating diabetic cardiomyopathy

**DOI:** 10.1007/s00210-024-03152-1

**Published:** 2024-05-22

**Authors:** Lamiaa Mohamed Mahmoud, Aya Aly Ashraf Abdel Mageed, Jackline Moawad Saadallah, Mira Farouk Youssef, Liala Ahmed Rashed, Hania Ibrahim Ammar

**Affiliations:** 1 Department of Physiology, Faculty of Medicine, Cairo University, Giza, Egypt; 2 Department of Histology, Faculty of Medicine, Cairo University, Giza, Egypt; 3 Department of Biochemistry, Faculty of Medicine, Cairo University, Giza, Egypt

**Keywords:** Diabetic cardiomyopathy, Stem cells, Anakinra, Interleukin 1β, Caspase1

## Abstract

Diabetic cardiomyopathy (DCM) is a serious common complication of diabetes. Unfortunately, there is no satisfied treatment for those patients and more studies are in critical need to cure them. Therefore, we aimed to carry out our current research to explore the role of two novel therapeutic approaches: one a biological drug aimed to block inflammatory signaling of the IL 1beta (IL1β) axis, namely, anakinra; the other is provision of anti-inflammatory regenerative stem cells. Wistar male rats were allocated into four groups: control group: type 2 diabetes mellitus (DM) induced by 6-week high-fat diet (HFD) followed by a single-dose streptozotocin (STZ) 35 mg/kg i.p., then rats were allocated into: DM: untreated; DM BM-MSCs: received a single dose of BM-MSCs (1 × 10^6^ cell/rat) into rat tail vein; DM-Anak received Anak 0.5 μg/kg/day i.p. for 2 weeks. Both therapeutic approaches improved cardiac performance, fibrosis, and hypertrophy. In addition, blood glucose and insulin resistance decreased, while the antioxidant parameter, nuclear factor erythroid 2–related factor 2 (Nrf2) and interleukin 10 (IL10), and anti-inflammatory agent increased. Furthermore, there is a significant reduction in tumor necrosis factor alpha (TNFα), IL1β, caspase1, macrophage marker CD 11b, inducible nitric oxide synthase (iNOS), and T-cell marker CD 8. Both Anak and BM-MSCs effectively ameliorated inflammatory markers and cardiac performance as compared to non-treated diabetics. Improvement is mostly due to anti-inflammatory, antioxidant, anti-apoptotic properties, and regulation of TNFα/IL1β/caspase1 and Nrf2/IL10 pathways.

## Introduction

Cardiovascular disorder is a serious complication in diabetic patients, with 2–5 times higher risk of developing heart failure (HF) than age-matched non-diabetic patients, independent of other comorbidities (Park 2021). Diabetic cardiomyopathy (DCM) is a distinct condition defined by the presence of myocardial dysfunction in absence of coronary atherosclerosis (Jia et al. 2018). Myocardial hypertrophy and fibrosis associated with diastolic dysfunction and later systolic dysfunction are hallmarks of such abnormality.

One possible mechanism of DCM pertains to uncontrolled activation of the nuclear factor kappa b/tumor necrosis factor alpha (NF-κΒ/TNFα) signaling mediated by excessive accumulation of reactive oxygen species (ROS) and advanced glycation end products thus pointing to a critical role of inflammation (Jin Joo Park 2021). DM is currently known as a disease of low-grade inflammation. In this regard, it is worth noting that NF-κB/TNFα is an upstream signaling pathway (Pickering et al. 2018), and interleukin 1β (IL 1β) axis is a cornerstone in the pathophysiology of DM. Besides playing a central role in innate immunity, unrestrained activation of IL 1β induces apoptosis, abnormal calcium handling, and hypertrophy of cardiomyocytes in DCM. Adipose tissue is the main source of inflammatory cytokines initiating a positive immune feedback, myocardial metabolic dysregulation, and activation of damage associated molecular patterns that further induce cardiac inflammatory responses (Liu et al. 2021; Helou et al. 2019; Yu et al. 2021; Peiró et al. 2017; Higashikuni et al. 2013).

Despite the gained success over glycemic control in diabetic patients, no therapies have effectively reversed DCM. In this context, targeted anti-inflammatory drugs have shown promising results in reversing diabetic complications (Silva et al. 2022; Agrawal and Kant 2014; Pappachan et al. 2013; Pollack et al. 2016). One of these anti-inflammatory drugs is anakinra (Anak) which is IL1β receptor blocker and plays a central role in IL-1 therapeutics. It is initially developed for rheumatoid arthritis and was confirmed to be safe and accompanied by protective cardiovascular effects in these patients (Ikonomidis et al. 2008). Recently, it has been more effective in the management of Covid pandemic with 77% reduced mortality and lesser complications (Liu et al. 2023). In the context of DM, the growing body of experimental data indicates the central role of Anak in T1DM and T2DM as well. In preclinical models of DM, Anak suppressed vascular inflammation, endothelial dysfunction, and the expression of adhesion molecules (Peiró et al. 2017). In addition, Anak reduced CRP, IL-6 levels, and C-peptide secretion in T2DM patients; this effect persisted over 39weeks following withdrawal of Anak (Pollack et al. 2016; Larsen et al. 2007). However, the potential cardiovascular benefits of Anak in DM have not been adequately explored yet.

Adding to the multitude of therapeutic approaches, bone marrow mesenchymal stem cell (BM-MSC) therapy has provided promising data in ameliorating DCM (Huang et al. 2022). As they possess multidirectional differentiation potential and self-renewal. In addition, BM-MSCs have the privilege over other stem cells of low immunogenicity and considerable immunomodulatory potential by interacting with the innate and adaptive immune systems (Huang et al. 2022). Moreover, stem cell therapy is safe, feasible, and has been tested repeatedly in different clinical trials, used now in many diseases as cancer and acute graft versus host disease due to their inhibitory effects on the proliferation and cytotoxic activity of immune system cells. Many studies have shown good tolerability to MSCs, with no adverse effects, and encouraging partial or complete response rates (Amorin et al. 2014; Mousaei Ghasroldasht et al. 2022). To date, there are no clinical trials testing the efficacy of Anak or MSC therapy in DCM and their role in suppressing TNFα/IL lβ and immune cell activation in DCM. Therefore, in the absence of globally effective therapy for DCM, we conducted this study to validate the potential role of these two novel therapeutic approaches.

## Materials and methods

### Ethics

The Institutional Animal Care and Use Committee of Cairo University approved the current research in accordance with ARRIVE guidelines and EU Directive 2010/63/EU guidelines for animals. The approval number is ID: CU III F 28 20.

### Chemicals

Anak was provided by Swedish Orphan Biovitrum AB, Sweden, and streptozotocin (STZ) was from Sigma Aldrich CO, USA. ELISA kits of insulin (Catalog #E2051Hu, Sunlong Biotech, China), IL10 (Catalog # SL0415Ra, Sunlong, Biotech, China), TNFα (Catalog # SL0722Ra, Sunlong, Biotech, China), IL1β polyclonal rabbit anti-rat antibody, Nrf2 polyclonal anti-rat antibody, and caspase1 polyclonal rabbit anti-rat antibody were from Biospes Co., China, with catalog numbers (Catalog #YPA1070), (Catalog# YPA1621), and (Catalog # YPA2348), respectively. TNFα polyclonal rabbit anti-rat antibody was from Elabscience, USA (Catalog # E-AB-33121). iNOS polyclonal rabbit anti-mouse antibody was from Abcam Medical, Cambridge, USA (Catalog # ab15323). CD11b rabbit monoclonal antibody was from Abcam, USA (Catalog # ab133357 ). CD8 beta rabbit monoclonal antibody was from Abcam, USA (Catalog # ab228965).

### In vivo studies

The current study was carried out at the Physiology and Biochemistry Departments, Faculty of Medicine, Cairo University. A total of 32 male Wistar rats aged 6–7 weeks, and their body weights ranged from 150 to 160 g were included. Rats were kept in the animal care and housed in the chip-bedded cages (3 rats/cage) at room temperature (25 ± 2 °C), humidity of 65–69%, and maintained on 12 h light/dark cycle. The rats were provided with high-fat diet (HFD) enriched rat chow and water ad libitum.

### Animal grouping and experimental protocol

For induction of DM type II, rats received HFD (57% fat, 25% protein, 17% carbohydrate) for 6 weeks then STZ (35mg/kg) single intraperitoneal injection (Gheibi et al. 2017).

Preparation of STZ: it was dissolved in freshly prepared citrate buffer (pH adjusted to 4.5); the solution was prepared immediately before injection and used within 5–15 min of dissolution. As STZ is sensitive to light, tubes were covered with aluminum foil. Rats were injected with STZ after 6-h fasting period (Gheibi et al. 2017). To prevent hypoglycemia and mortality during the first 48 h, 10% glucose solution was given orally to diabetic rats (Gheibi et al. 2017). Control rats were kept in the same conditions and had free access to food and water till the end of the study period (16 weeks).

After 1 week, fasting blood glucose was measured and those with a fasting blood glucose level >150 mg/dl and non-fasting blood glucose levels >250 mg/dl were considered diabetic (Gheibi et al. 2017). Eight weeks after STZ injection, diabetic rats were randomly divided into diabetic non-treated (DM); diabetic treated with MSCs (DM BM-MSCs) (injected with a single dose of (1 × 10^6^ BM-MSCs per rat) suspended in phosphate-buffered saline (PBS) into rat tail vein (Mendelsohn and Larrick 2017) then kept for 2 weeks till the end of study period; diabetic treated with Anak (DM-Anak) received it in a dose of 0.5 μg/kg/day by intraperitoneal injection for 2 weeks (Amin et al. 2012).

### Echocardiography

Rat echocardiography was performed at baseline, after 6 weeks of HFD, after 8 weeks of STZ injection and at the end of the study. Rats were anesthetized with ketamine hydrochloride (25 mg/kg) single intraperitoneal injection. After shaving the chest, rats were placed in supine position, and the transducer was placed directly on chest wall. Echocardiograms were recorded with an echocardiography system equipped with a 9-MHz phased-array micro convex transducer and animal echocardiography machine (Mindray D Pvet 20). Two-dimensional short axis views of left ventricle and M-mode tracings were recorded through anterior and posterior LV walls at the level of the papillary muscles. The following parameters were measured: end diastolic diameter (LVEDD) in centimeter, end systolic diameter (LVESD) in centimeter, fractional shortening (FS%), and ejection fraction (EF%). FS% was calculated using Gibson’s equation: FS% = (LVEDD-LVESD/LVEDD) × 100. Diastolic function was also assessed using Doppler across mitral valve for determination of peak early diastolic filling velocity (*E* velocity) in cm/s, late diastolic filling velocity (*A* velocity) in cm/s, their ratio (*E*/*A* ratio), and the deceleration time (DT) in millisecond of early diastolic filling wave.

### In vitro studies

#### Isolation and propagation of MSCs

Bone marrow was isolated from age matched healthy rats. Under sterile conditions, femurs and tibiae were excised, and connective tissue attached to bones was removed with special attention. Bone marrow was harvested by flushing tibiae and femurs with Dulbecco’s modified Eagle’s medium (GIBCO, USA) supplemented with 10% fetal bovine medium (GIBCO, USA). Nucleated cells were isolated with a density gradient (Ficoll/Paque (Pharmacia)) and resuspended in complete culture medium supplemented with 1% penicillin-streptomycin (GIBCO, USA). Cells were incubated at 37 °C in 5% humidified CO_2_ for 24 h; the nonadherent cells were then removed and washed out using sterile PBS. The media were changed every 3 days, and cells from passage 3 were taken for the study (Ammar et al. 2021; Mendelsohn and Larrick 2017).

#### Immunotyping

Using flow cytometric analysis, expression of MSC markers was quantified. Adherent cells (at third passage) were trypsinized, adjusted to 1 × 10^6^ cells/ml. Then, 1 × 10^5^ cells were incubated with 10 μl of monoclonal antibodies: antibodies against CD29, CD34, CD45, and CD90 (Beckman coulter CO, USA) at 4 °C in the dark. Same species isotypes were used as negative control. Twenty minutes after incubation, 2 ml PBS containing 2% FCS solution were added to tubes of monoclonal treated cells. Mixtures were centrifuged 5 min at 2500 rpm then discarding resuspending cells in 500 μl PBS containing 2% FCS was done. Cell analysis was assessed by CYTOMICS FC 500 Flow Cytometer (Beckman coulter CO, FL, USA), analyzed by the CXP Software version 2.2.

#### Labeling of stem cells with PKH26 dye and detection

MSC cells were harvested and labeled with PKH26 red fluorescent linker dye (2 × 10^−6^ M PKH26 dye), and 1 × 10^7^ cells/ml in a 2-ml total volume was stained according to Sigma protocol steps (Sigma, St. Louis, MO). PKH26 is considered a good indicator as it is stable and can be visualized in ex vivo cell samples up to 100 days. In in vivo studies, its long-term stability and even distribution in dividing cells make it a good choice. Also, labeled cells retain both biological and proliferating activity (Shao-Fang et al. 2011). After 2 weeks of cell implantation, sections were assessed, and cell number was quantified .

### Histological studies

Cardiac tissues were cut longitudinally along the long axis of the ventricles to examine the whole ventricular chambers and immediately preserved in PBS 10% (pH 7.4) for 24 h. Heart tissues were dehydrated, clarified, and then embedded in paraffin.

#### Detection of transplanted MSCs homing in cardiac tissue

**Fig. 1:**
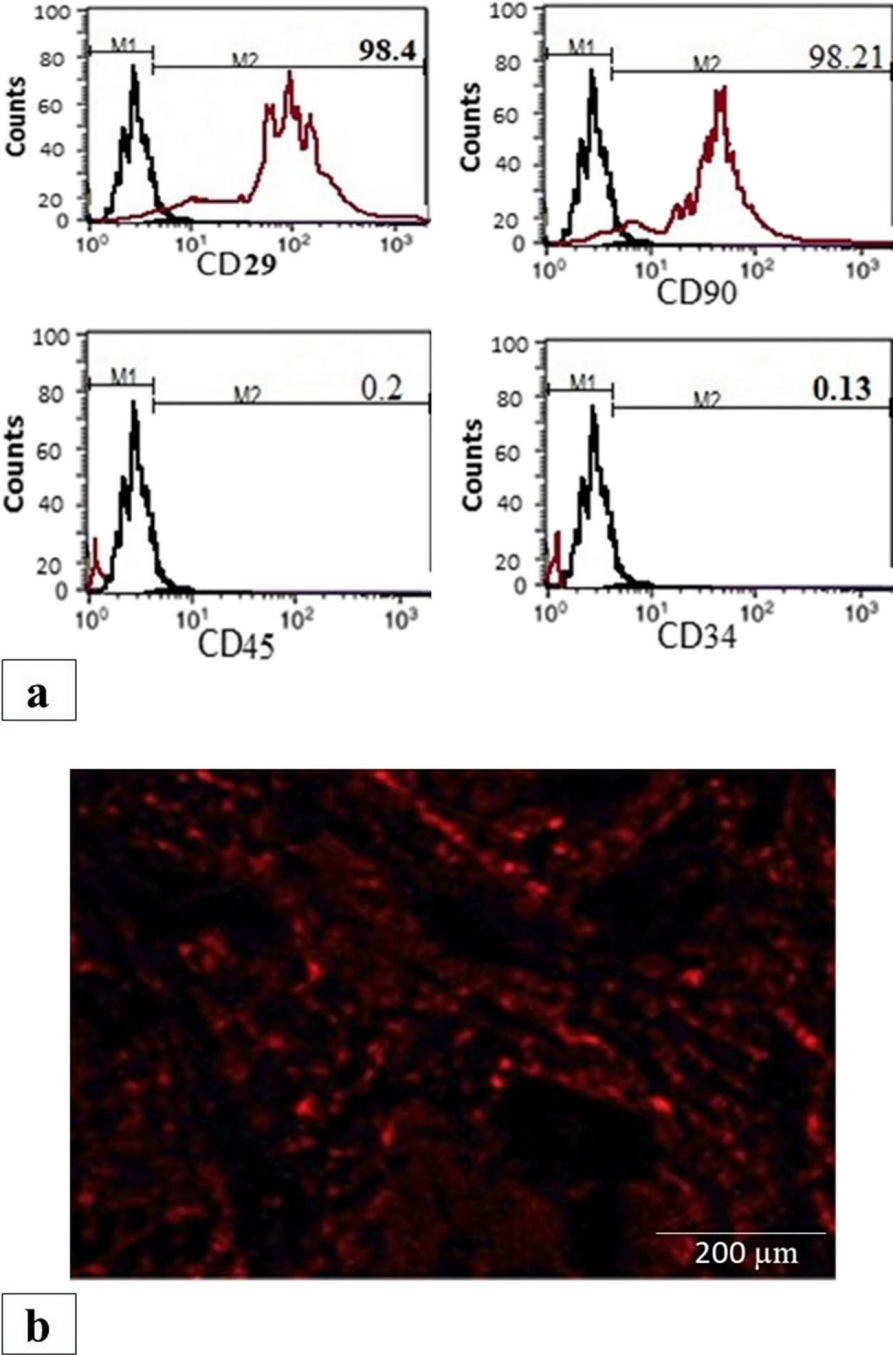
Identification and homing of BM-MSCs into cardiac tissue. **a** Immunofluorescence detection of BM-MSC markers (upper panel): cells were negative for the hematopoietic marker CD34 and CD45, while strongly positive for mesenchymal stem cell specific markers including CD29 and CD90. The red histograms represent antibody labeled cells, while the black histogram shows the profile of the isotype control. **b** Cardiac homing of transplanted BM-MSCs (lower panel): red fluorescent labeled MSCs in the DM BM-MSC group showing successful homing in the cardiac tissue after 2-week treatment

Before transplantation, MSCs were labelled with PKH26 dye using Sigma protocol (Sigma, St. Louis, MO). To count the number of transplanted cells, unstained sections were visualized using fluorescence microscope at 551 (excitation) and 567 (emission) wavelengths. The detected count was 57 at magnification 400× **(**Fig. 1**).**

#### Hematoxylin and eosin staining

For microscopical examination, 5-μm slices were cut and mounted on clean glass slides coated with Mayer’s egg albumin and stained with hematoxylin and eosin.

- Masson’s trichrome staining: This stain was used to selectively stain CT fibers, namely, collagen. Standard Masson’s trichrome procedure stains collagen blue, muscle tissue red, and nuclei dark brown. Percent area of collagen fibers was calculated using the Leica Qwin 500 LTD computer-assisted image analysis software (Cambridge, UK) (Ammar et al. 2015).
- Immunohistochemistry

Immunohistochemical staining was performed using rat sensitive primary polyclonal antibodies for TNFα. IL1β, Nrf2, iNOS, and caspase1 at working dilutions of 1:100 for TNFα, iNOS, and 1/40 for the remaining antibodies. Cardiac sections 5 μm were mounted on poly l-lysine-coated slides. After blockage of the endogenous peroxidase activity, the sections were placed in 0.01 mol/l citrate buffer in a microwave for 5 min for antigen retrieval; sections were then incubated in 1% BSA dissolved in PBS for 30 min at 37 °C. This was followed by incubation with the primary antibodies. The goat anti-rabbit IgG H&L (HRP) (E-IR-R211) served as the secondary antibody. Then, the sections were incubated with diaminobenzidine tetrahydrochloride, counterstained with Mayer’s hematoxylin, dehydrated, cleared, and mounted by DPX. Negative controls were processed via omission of the primary antibodies.

### Biochemical analysis of blood samples

At baseline and every 1 week starting from day zero following induction and verification of DM, fasting blood samples were obtained from rat tail vein for measuring blood sugar and serum insulin. At the end of study, animals were sacrificed using overdose of phenobarbital 150 mg/kg intraperitoneal injection for euthanasia. Blood samples were withdrawn from all groups for blood glucose and ELISA measurements of serum insulin, IL10 and TNFα. This was performed using Sandwich-ELISA method as provided by the kits respectively.

Calculation of HOMA-IR:

Homeostasis model assessment of insulin resistance (HOMA-IR) ((fasting insulin (U/l) × fasting glucose (mmol/L)/22.5) was calculated to all groups (Okoduwa et al. 2017).

Conversion factor: Insulin (1U/l = 7.174 pmol/l) and blood glucose (1 mmol/l = 18 mg/dl)

### Evaluation of CD11b and anti CD8 using Western blotting

Cardiac tissue from the control and treated groups was washed with PBS, lysed in lysis buffer. After centrifugation, the supernatant was prepared as a protein extracts. Then electrophoresis, immunoblotting, and protein detection were done for the macrophage marker CD11b and the T cell marker CD8 using anti CD11b and anti CD8 antibodies.

Band intensity was analyzed by ChemiDocTM imaging system with Image LabTM software version 5.1 (Bio-Rad Laboratories Inc., Hercules, CA, USA). The results were expressed as arbitrary units after normalization for β-actin protein expression.

### Statistical analysis

Data were analyzed by GraphPad Prism version 5 using mean and standard deviation (SD), and comparisons between groups were done using analysis of variance (ANOVA) then Tukey’s multiple comparison test. A probability value *P* values less than 0.05 were considered as statistically significant**.**

## Results

### Evaluation of glycemic control of BM-MSCs or Anak

The three parameters measured for testing the glycemic control were fasting blood glucose (FBG), serum insulin, and HOMA IR test. At baseline, all groups showed similar values. The DCM-induced group showed significant increase of all of these parameters compared to the control group. However, they significantly reduced in the treated groups compared to the untreated DCM group (Table 1).

**Table 1:** Fasting blood glucose, serum insulin, and HOMA-IR

Groups	Control	DM	DM-MSC	DM-Anak
FBG (gm/dl)	**120.3 ± 4.5**	**464.3 ± 51.73acd**	**212.7 ± 11.02ab**	**163.3 ± 22.55b**
Insulin(U/L)	**7.42 ± 1**	**17.94 ± 0.76acd**	**15.60 ± 0.4abd**	**13.12 ± 1.31abc**
HOMA IR	**3.17 ± 0.99**	**18.62 ± 2.15acd**	**7.58 ± 0.49abd**	**5.29 ± 0.36abc**

Results of study were for eight observations compared as mean ± SD. Significance was if *p* < 0.05. a: Statistically significant compared to the control group. b: Statistically significant compared to the diabetic untreated group (*p* < 0.05). c: Statistically significant compared to the diabetic group treated with stem cell therapy (BM-MSCs). d: Statistically significant compared to the diabetic group treated with anakinra (Anak)

### Evaluation of cardiac functions upon administration of BM-MSCs or Anak

**Fig. 2:**
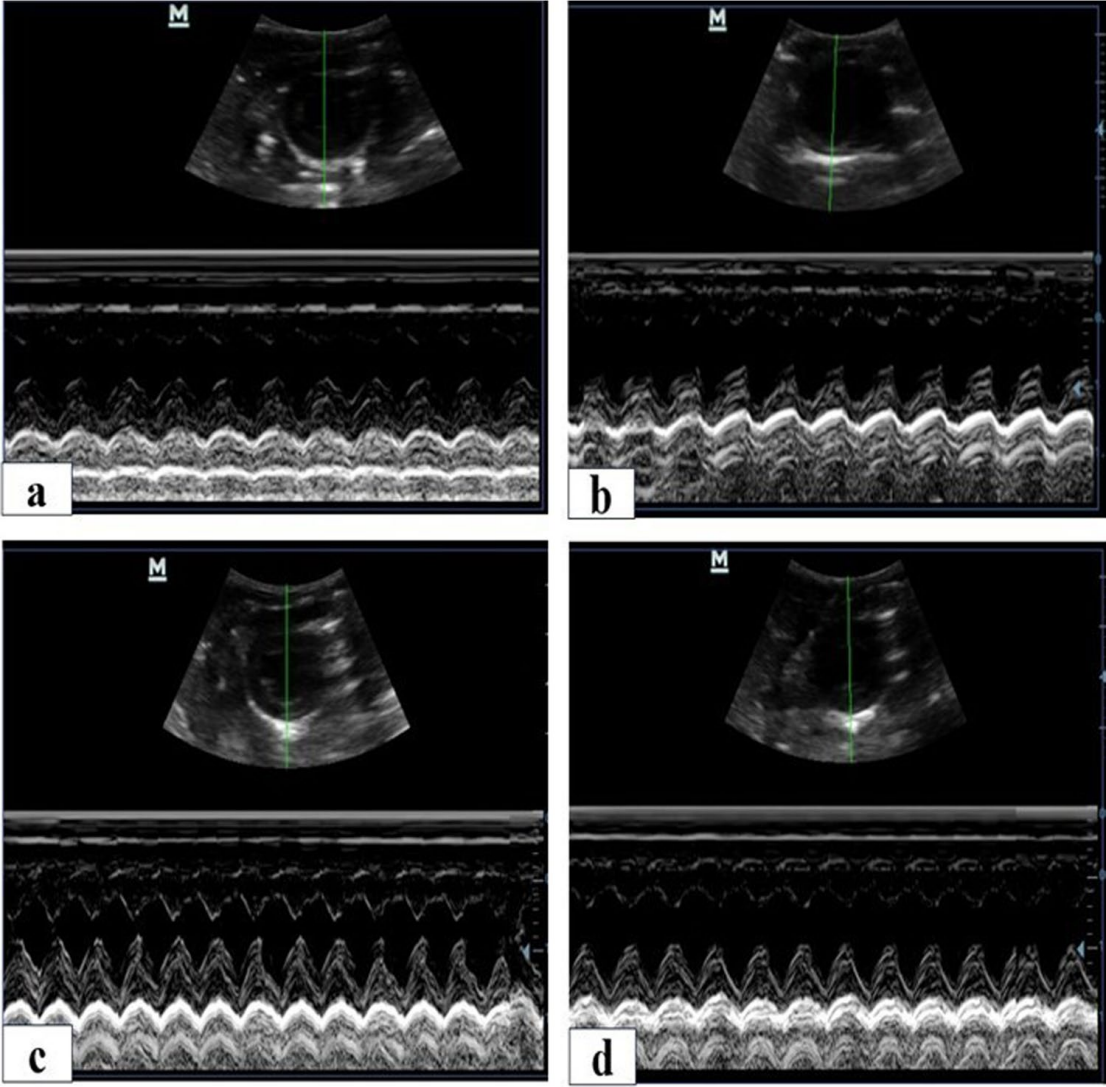
Ejection fraction, fractional shortening, and left ventricular end diastolic diameters calculated from diabetics treated and non-treated rats at the end of study. Short-axis views were obtained from all studied groups at the end of the study. Representative echo images for control (**a**), DM( **b**), DM-BM MSCs (**c**), and DM-Anak (**d**). At the eight week following STZ injection, all groups showed significant reduction in EF and FS compared to controls. The DM group remained lower till the end of the study. Those receiving stem cells or Anak showed normalization of their EF and FS with significantly higher values than the DM group. Diabetes resulted in elevated LVEDD recorded at the eight week. In the treated groups, LVEDD was reduced to be significantly lower than non-treated diabetics but remained elevated than controls. Results of study were for eight observations compared as mean ± SD. Significance was if *p* < 0.05. **a** Statistically significant compared to the control group, **b** statistically significant compared to the diabetic untreated group (*p* < 0.05). **c** Statistically significant compared to the diabetic group treated with stem cell therapy (BM-MSCs). **d** Statistically significant compared to the diabetic group treated with anakinra (Anak)

When recording echocardiographic data, all groups had the same baseline values. At 6 weeks HFD, there was no change from baseline data. After 8 weeks of STZ, induction of DCM resulted in deterioration in EF, FS, and increase in LVEDD. Interestingly, either BM-MSCs or Anak reversed the deterioration in cardiac functions. EF and FS were significantly higher than untreated diabetics and comparable to controls. All diabetic groups showed signs of dilated cardiomyopathy indicated by elevated LVEDD compared to controls which partially improved upon treatment. BM-MSCs or Anak therapy showed significant reduction in LEVDD compared to the DM group (Fig. 2) (Table 2).

**Table 2:** Echocardiographic and doppler parameters

Groups	Control	DM	DM-MSC	DM-Anak
LVEDD (cm) end study	**0.55 ± 0.007**	**0.80 ± 0.04acd**	**0.69 ± 0.04abd**	**0.62 ± 0.006abc**
EF % end study	**87.28% ± 2.19**	**73.42% ± 2.4acd**	**81.43% ± 3.8ab**	**82.42% ± 2.63b**
FS% end study	**52.25% ± 3.86**	**39.91% ± 1.6acd**	**48.62% ± 5.28bd**	**56.67% ± 1.53bc**
E wave(cm/s) end study	**65.97 ± 5.19**	**59.50 ± 11.53cd**	**77.56 ± 4.83b**	**75 ± 3.24b**
A wave (cm/s) end study	**32.34 ± 4.7**	**53.19 ± 5.12ad**	**58.27 ± 13.28ad**	**30.16 ± 3.36bc**
E/A end study	**2.06 ± 0.25**	**1.17 ± 0.29ad**	**1.38 ± 0.30ad**	**2.5 ± 0.31bc**
DT (ms) end study	**46.80 ± 2.39**	**62.20 ± 2.49acd**	**46.20 ± 5.31b**	**40.33 ± 4.04b**

Results of study were for eight observations compared as mean ± SD. Significance was if *p* < 0.05. a: Statistically significant compared to the control group. b: Statistically significant compared to the diabetic untreated group (*p* < 0.05). c: Statistically significant compared to the diabetic group treated with stem cell therapy (BM-MSCs). d: Statistically significant compared to the diabetic group treated with anakinra (Anak)

### Diastolic dysfunction with or without BM-MSCs or Anak therapy

**Fig. 3:**
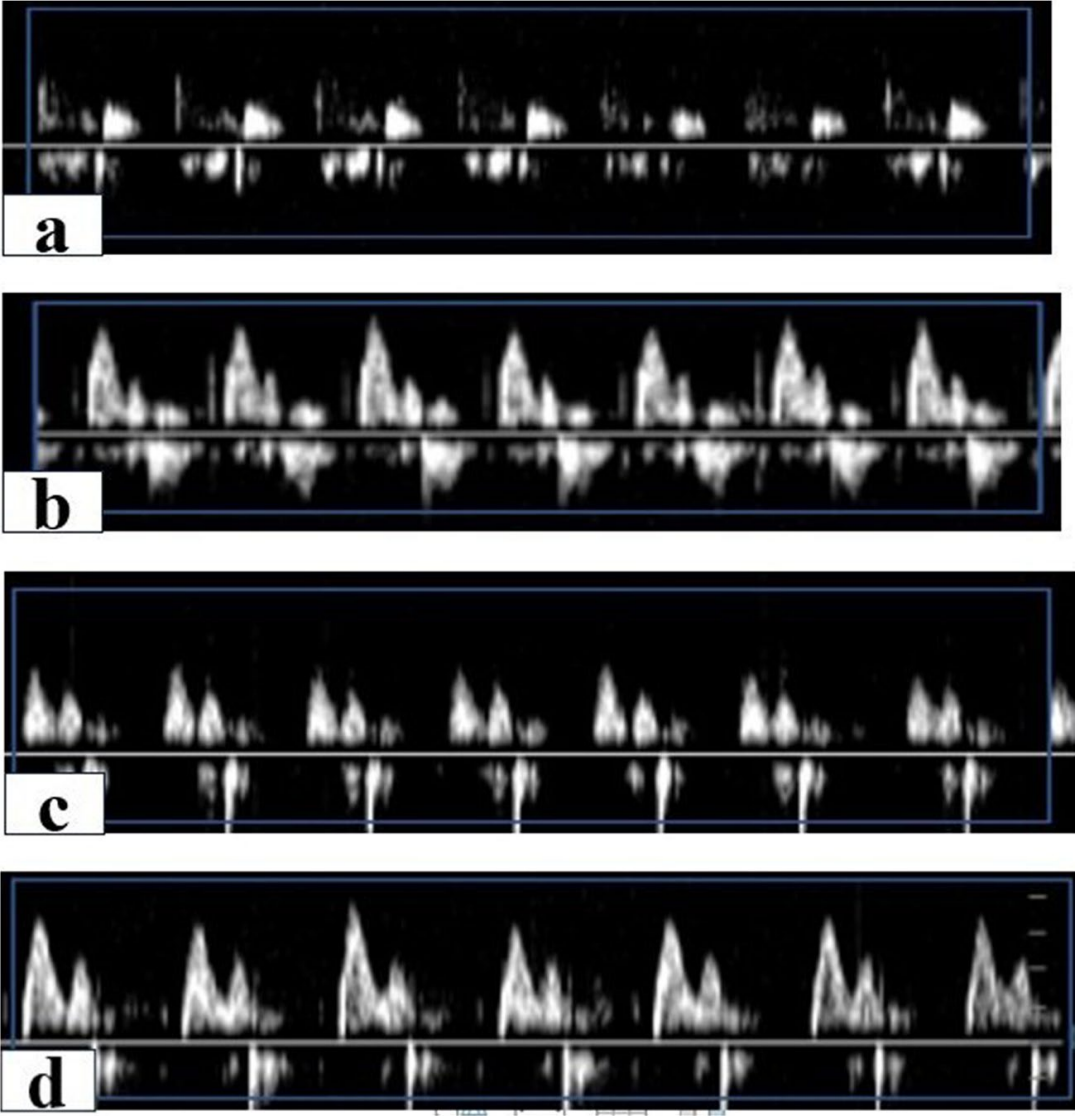
Doppler imaging for mitral inflow velocity in diabetic non-treated and treated rats at the end of study. Representative images for Doppler mitral inflow velocities for control (**a**), DM (**b**), DM-BM MSCs (**c**), and DM-Anak (**d**). E, early mitral inflow velocity; A, late mitral inflow velocity; E/A, ratio and deceleration time were recorded. Eight weeks after STZ, all diabetic rats (non-treated and treated) showed a decrease in E/A ratio associated with increase in A velocities and a decrease in E velocities together with prolongation of deceleration time, indicating impaired relaxation and early diastolic dysfunction. These changes persisted in DM group till the end of the study. Treatment with BM-MSCs or Anak resulted in enhanced diastolic function and normalization of E/A ratio. Results of study were for eight observations compared as mean ± SD. Significance was if *p* < 0.05. **a** Statistically significant compared to the control group, **b** Statistically significant compared to the diabetic untreated group (*p* < 0.05). **c** Statistically significant compared to the diabetic group treated with stem cell therapy (BM-MSCs). **d** Statistically significant compared to the diabetic group treated with anakinra (Anak)

At baseline, there was no significant difference in mitral inflow velocities among all groups. Induction of diabetes caused signs of early diastolic dysfunction indicated by a significant decrease in *E* velocity, increase in *A* velocity, reduced *E*/*A* ratio, and prolongation of deceleration time. BM-MSC and Anak showed improvement in diastolic dysfunction indicated by a significant increase in *E* velocity, decrease in *A* velocity, normalized *E*/*A* ratio, and deceleration time (Fig. 3) (Table 2).

### BM-MSCs or Anak improved degenerative and fibrotic changes

#### Hematoxylin and eosin

H&E-stained sections of the DCM group revealed degenerative changes in cardiac fibers. LS revealed areas of non-uniform fiber size, where some areas were thinned out. Other areas showed marked corrugation of outline and tears of fibers. Staining was heterogenous, with areas showing rarefaction of cytoplasm demonstrated as pale acidophilia, and others showed more dense staining. Demarcation line was the intercalated disc, and some nuclei were closed (dark). Intercalated discs were preserved. TS sections also expressed marked variation of fiber diameter. Localized areas showed enlarged dark fibers with peripheral displacement of nuclei. Vacuolations were evident and widespread inside many fibers. Small blood vessels were seen.

**Fig. 4:**
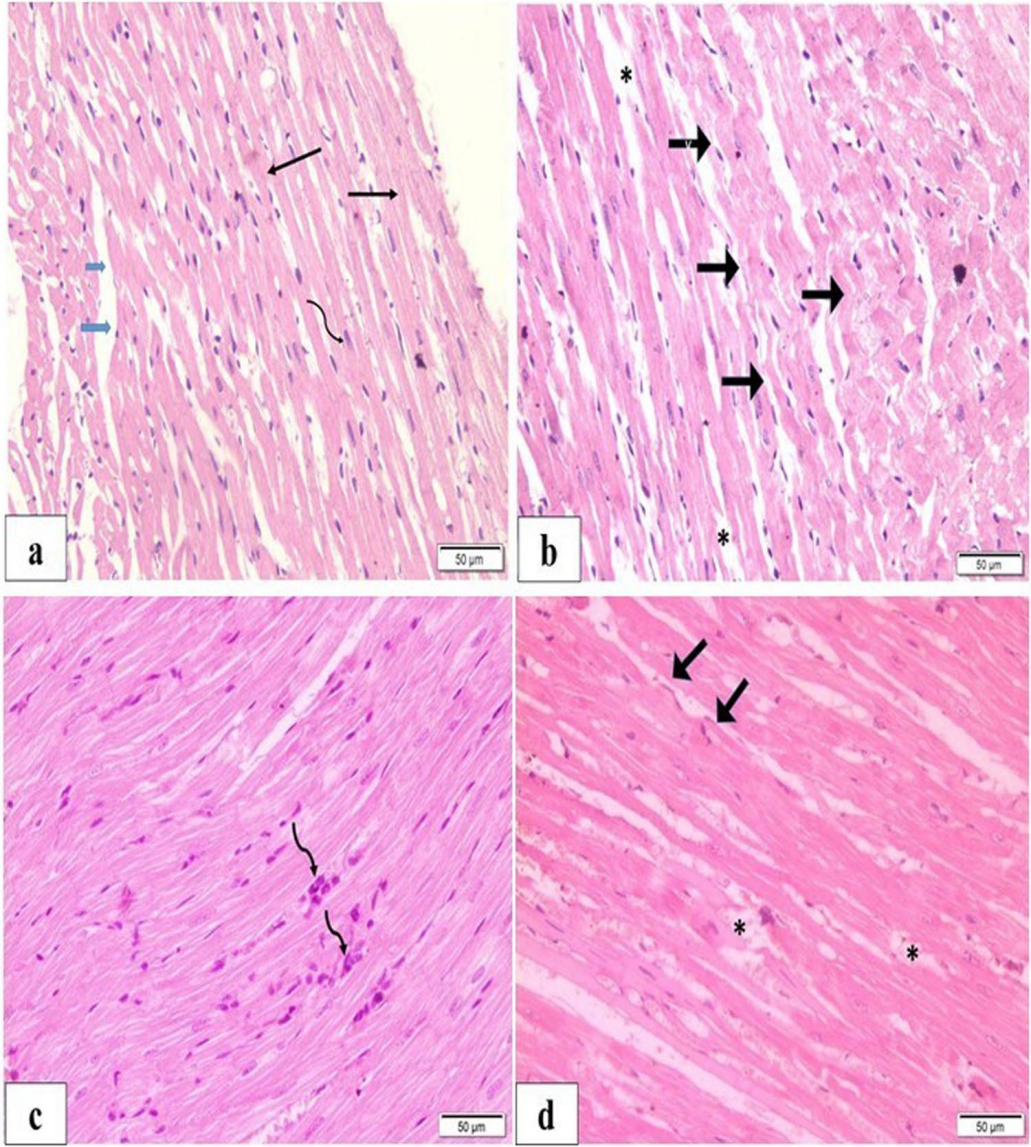
H&E staining in diabetic treated and diabetic non-treated rats. Representative H&E staining in controls (**a**) showing normal branching of fibers (black arrow), central nuclei (curved arrow), minimal connective tissue (blue arrow); diabetic non-treated (**b**) showing areas of corrugated outlines (black arrows) and muscle fiber loss of continuity (asterisk). In the treated groups (**c**, **d**), previous findings were improved compared to the DM group with less pathological changes. **c** Stem cell monotherapy showed some aggregated nuclei (curved arrows); **d** Anak showed some corrugations (thick black arrow) and some loss of continuity (asterisk)

The treated groups showed improvement compared to the DCM group. However, histological sections still showed pathological changes, but to a lesser extent. The BM-MSC and Anak groups showed that arrangement and staining of fibers were improved over wide areas. Connective tissue distribution was within normal; there were some areas of densely stained fibers, and other areas showed corrugation, and disruption of continuity with loss of striation and dark nuclei (Fig. 4).

#### Masson trichrome stain for connective tissue

**Fig. 5:**
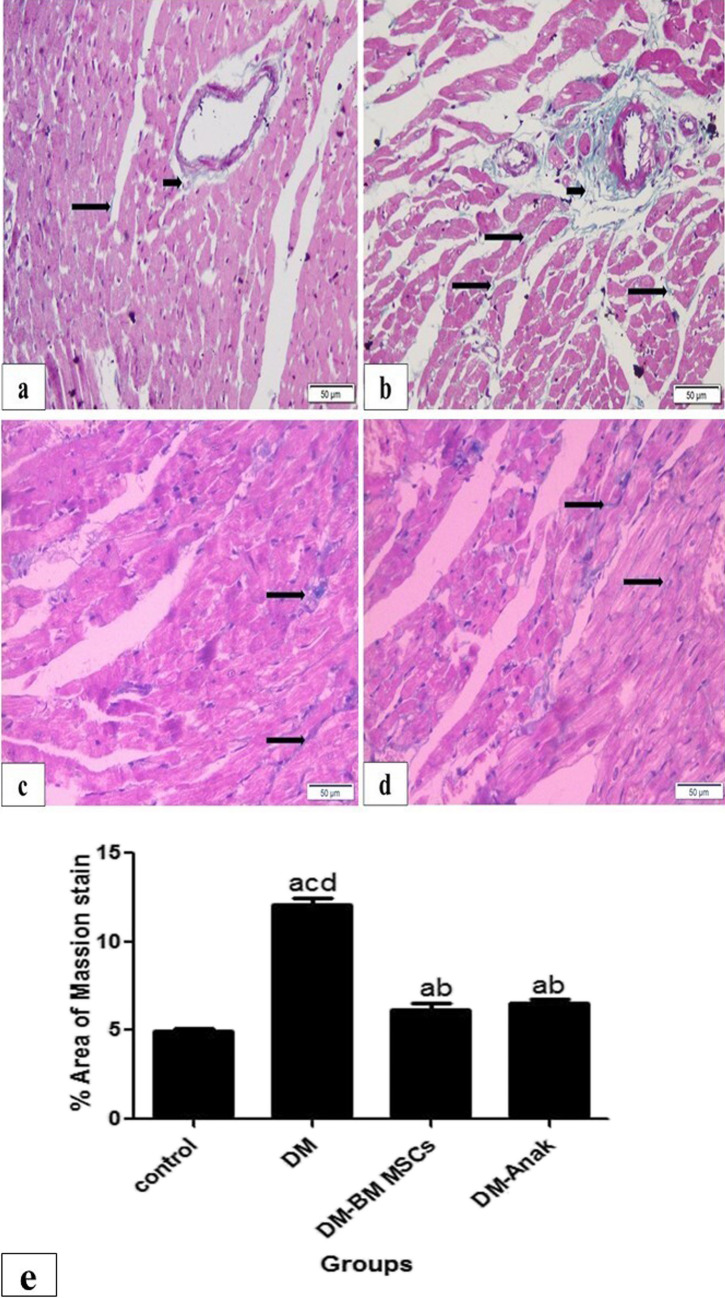
Masson trichrome staining in diabetic treated and diabetic non-treated rats. Masson trichrome stain for connective tissue (CT) in control group (**a**) showing normal minimal CT (light blue color) both interstitial (long arrow) and perivascular (short arrow); diabetic non-treated group (**b**) showing markedly increased CT evident both interstitial and perivascular. Monotherapy treated groups (**c**, **d**) showed an amount of CT that was more than the control group but less than the non-treated group. **e** Calculation of percent area of fibrosis: the DM group revealed significantly increased area % both interstitial and perivascular as compared to the control group. This percent was significantly reduced in treated groups compared to the diabetic untreated group, but still significantly higher than the control group. More improvement is obvious in Anak given group. Results of study were for eight observations compared as mean ± SD. Significance was if *p* < 0.05. **a** Statistically significant compared to the control group. **b** Statistically significant compared to the diabetic untreated group (*p* < 0.05). **c** Statistically significant compared to the diabetic group treated with stem cell therapy (BM-MSCs). **d** Statistically significant compared to the diabetic group treated with anakinra (Anak)

The DM group showed statistically significant increase in connective tissue area % in comparison to the control group that was evident both interstitial as well as perivascular. All the treated groups showed significant reduction in connective tissue area % compared to the DCM group, but still significantly higher than the control group (Fig. 5).

#### Evaluation of TNFα, IL1β, and Nrf2 immunoexpression

**Fig. 6:**
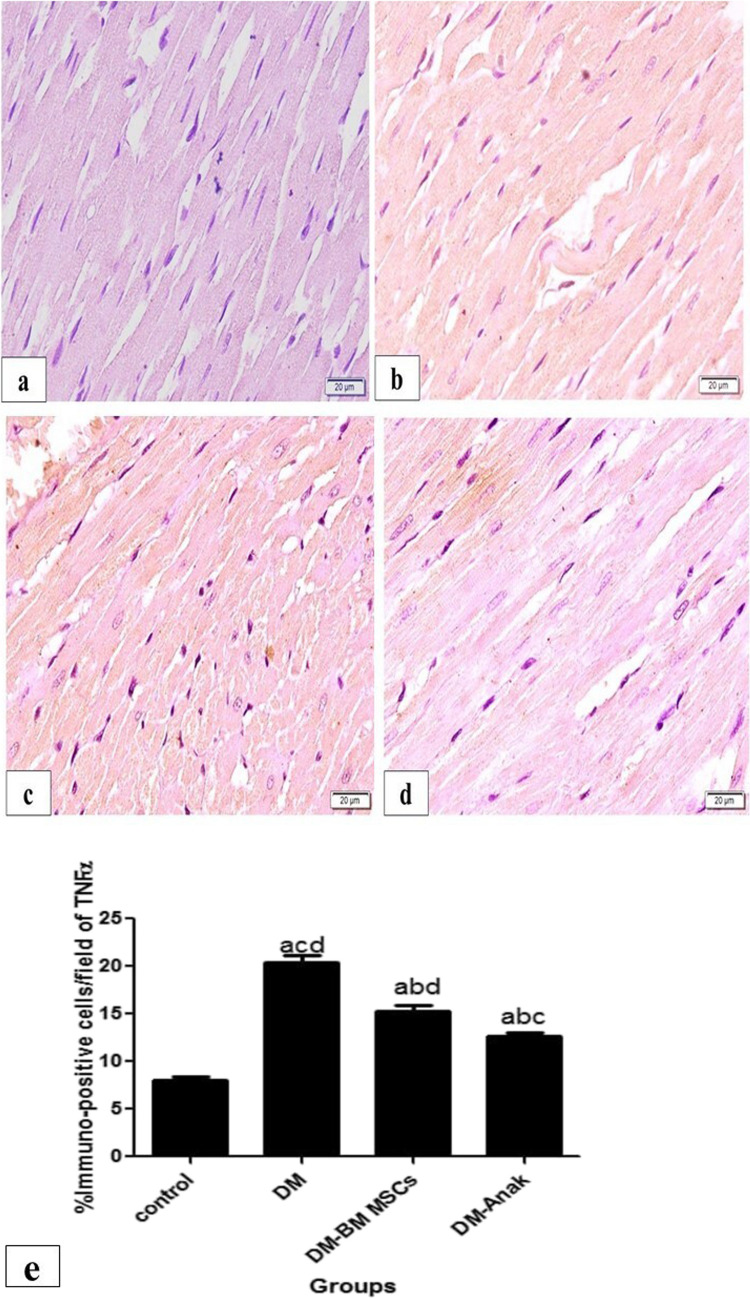
Immunostaining of TNFα: staining in the control group (**a**) was minimal, mostly granular, and homogenous in distribution; in the diabetic non-treated group (b), staining was markedly increased, taking most of the fibers. The treated groups (**c**. **d**) showed markedly increased staining compared to the control group, while they revealed less distributed staining compared to the diabetic non-treated group. The stem cell treated group (**c**) showed widespread granular reaction. The Anak treated group (**d**) showed more homogenous distribution, similar in pattern to control group. **e** Calculation of percent area: Immunostaining of TNFα significantly increased in the diabetic untreated group compared to the control group, but significantly decreased in the stem cell or Anak given groups compared to the diabetic untreated group. More improvement is obvious in the Anak given group. Results of study were for eight observations compared as mean ± SD. Significance was if *p* < 0.05. **a** Statistically significant compared to the control group. **b** Statistically significant compared to the diabetic untreated group (*p* < 0.05). **c** Statistically significant compared to the diabetic group treated with stem cell therapy (BM-MSCs). **d** Statistically significant compared to the diabetic group treated with anakinra (Anak)

**Fig. 7:**
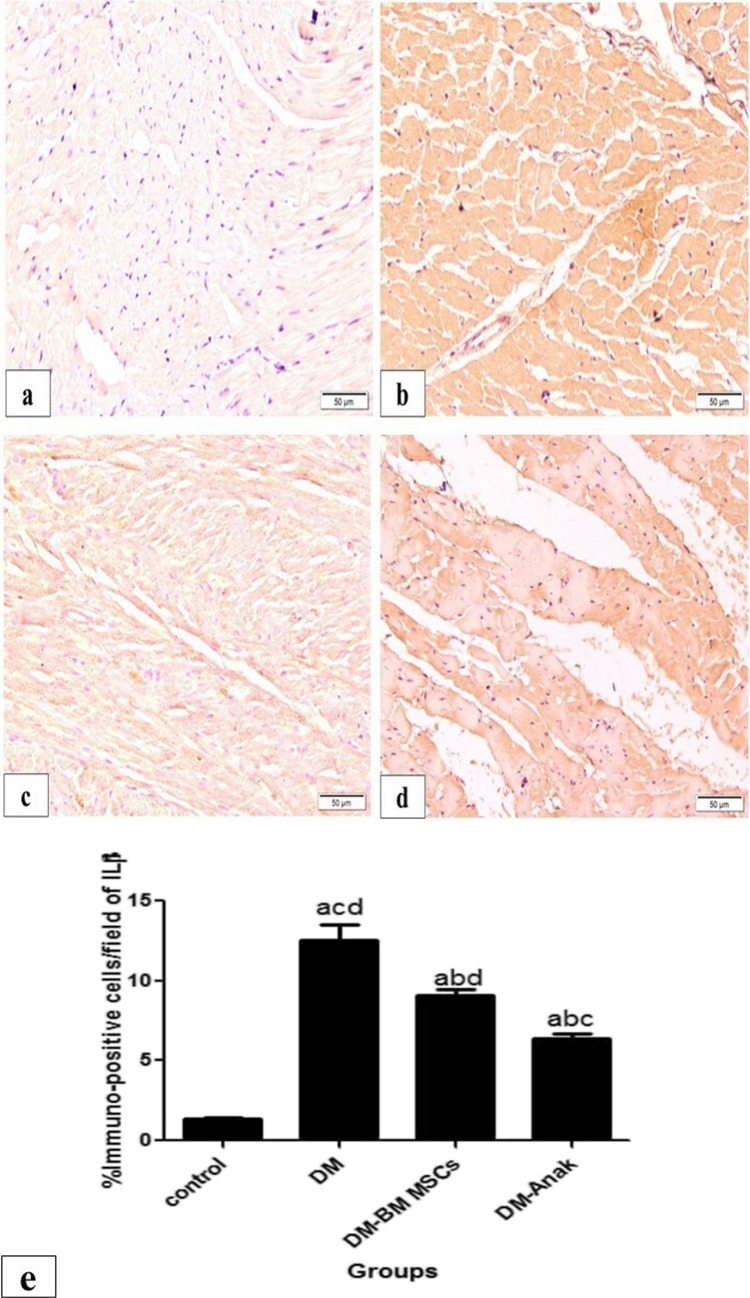
Immunostaining of IL1β. In the control group (**a**), reaction was minimal and homogenous; in the diabetic nontreated group (b), staining increased and was mostly also homogenous. The treated groups (**c**, **d**) revealed less staining compared to the diabetic non-treated group. The Anak treated group (**d**) showed marked improvement compared to the stem cell treated group. **e** Calculation of percent area: The diabetic untreated group showed significant increase in IL1β staining compared to the control group. However, IL1β significantly decreased in the treated groups compared to the diabetic untreated group. The Anak treated group showed more improvement. Results of study were for eight observations compared as mean ± SD. Significance was if *p* < 0.05. **a** Statistically significant compared to the control group. **b** Statistically significant compared to the diabetic untreated group (*p* < 0.05). **c** Statistically significant compared to the diabetic group treated with stem cell therapy (BM-MSCs). **d** Statistically significant compared to the diabetic group treated with anakinra (Anak)

Immunostaining for TNFα and IL1β was significantly increased in the DM group compared to the control group. However, staining was significantly lower in the MSC and Anak treated groups as compared to the DCM group (Figs. 6 and 7).

**Fig. 8:**
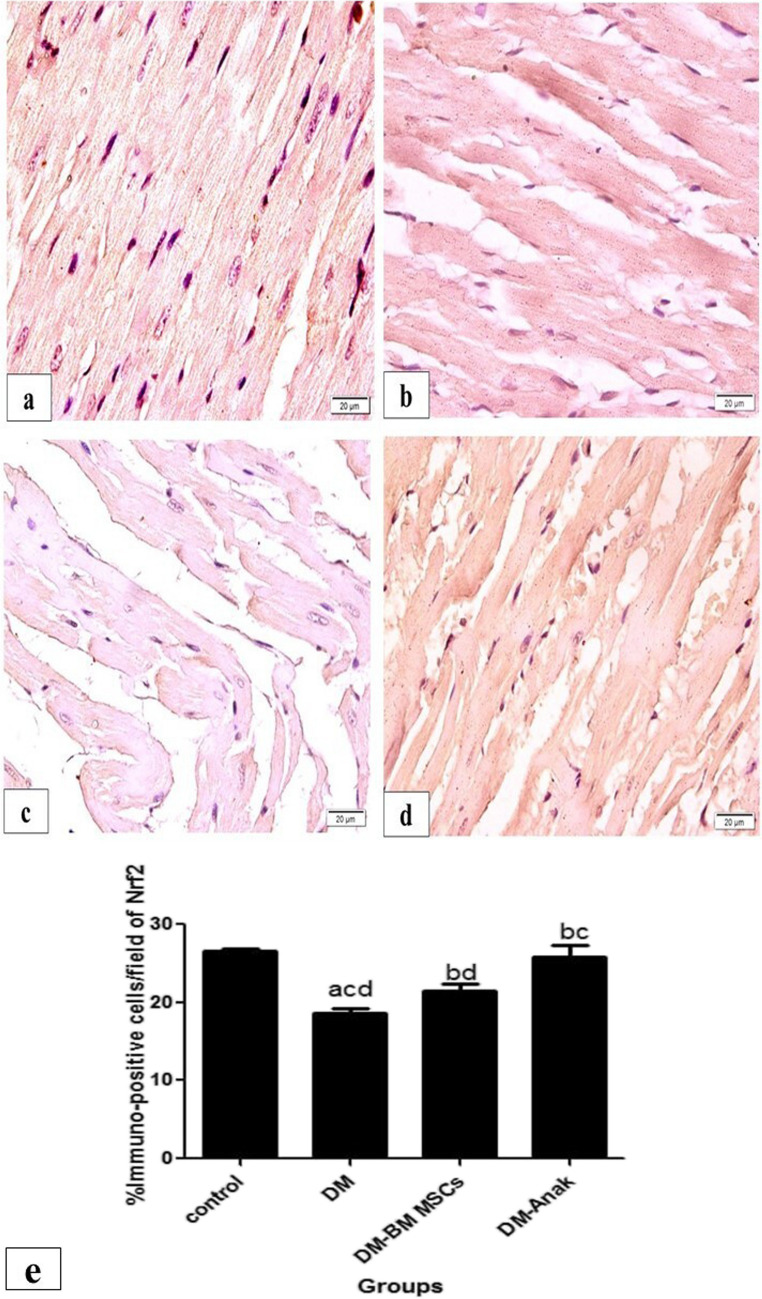
Immunostaining of Nrf2. The control group stained for Nrf2 (**a**) showed homogenous widespread staining, distributed inside muscle fibers. The diabetic non-treated group (**b**) showed less positive staining, with slightly irregular pattern. The treated groups (**c**, **d**) showed increased staining. e Calculation of percent area: Immunostaining of Nrf2 significantly decreased in the diabetic untreated group compared to control. However, Nrf2 significantly increased in the stem cell or Anak given groups compared to diabetic untreated group. More improvement is obvious in the Anak given group. Results of study were for eight observations compared as mean ± SD. Significance was if *p* < 0.05. **a** Statistically significant compared to the control group. **b** Statistically significant compared to the diabetic untreated group (*p* < 0.05). **c** Statistically significant compared to the diabetic group treated with stem cell therapy (BM-MSCs). **d** Statistically significant compared to the diabetic group treated with anakinra (Anak)

The DM group showed significantly less positive staining of the anti-inflammatory Nrf2 than the control group, with slightly irregular pattern. However, the treated groups showed a significant increase in NrF-2 staining compared to the DCM group. Staining was still less than control except for Anak that was of the same value as control. Staining was mainly located peripheral within the muscle fibers. It was noted that enlarged affected fibers showed markedly less staining (Fig. 8).

#### Pyroptotic caspase1 and iNOS immunoexpression

**Fig. 9:**
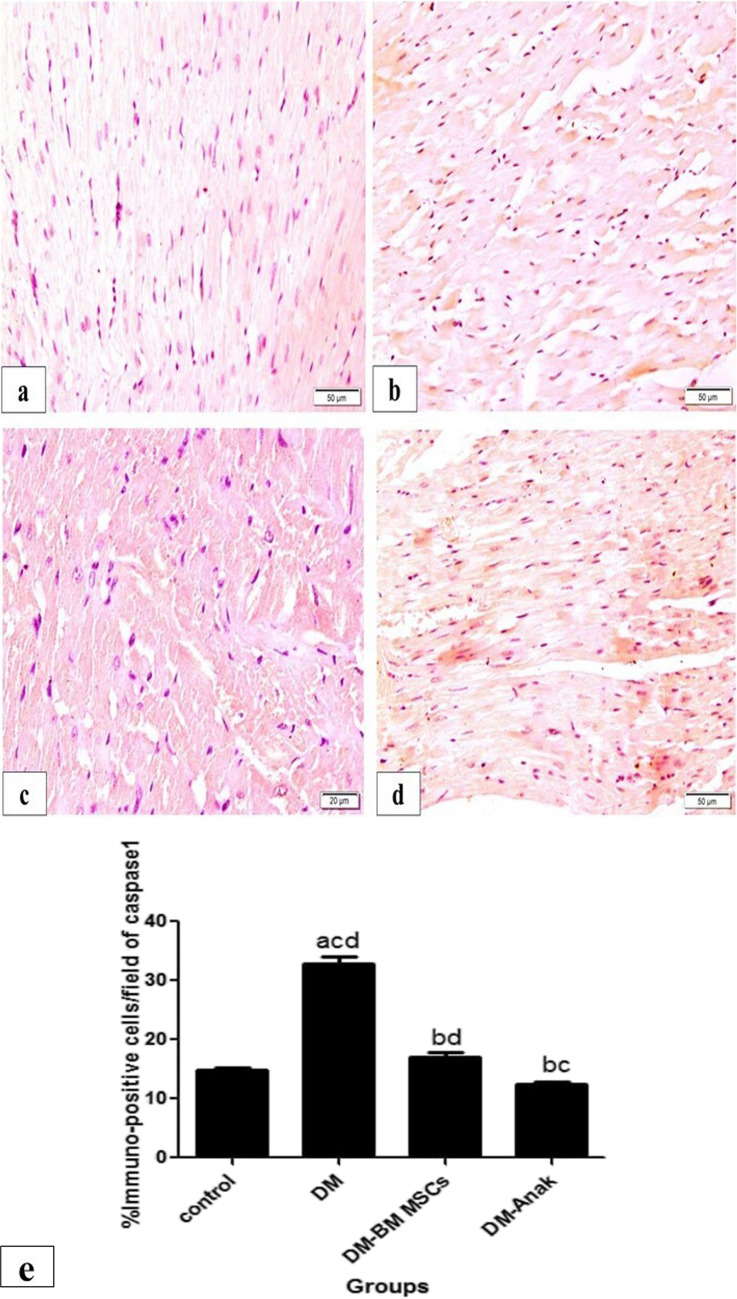
Caspase1 immunoexpression. Caspase1 immunoexpression of the control group (**a**) had homogenous little distribution of caspase1 immunostaining. Its expression obviously increased in the diabetic non-treated group. **b** Stem cell or Anak showed less distribution of immunostaining. **e** Calculation of percent area of caspase1: The diabetic non-treated group significantly increased immunostaining compared to the control group, while the treated groups had significantly reduced caspase1 immunostaining compared to the diabetic untreated group. This made these values near normal as there was no significance compared to control rats’ values. Results of study were for eight observations compared as mean ± SD. Significance was if *p* < 0.05. **a** Statistically significant compared to the control group. **b** Statistically significant compared to the diabetic untreated group (*p* < 0.05). **c** Statistically significant compared to the diabetic group treated with stem cell therapy (BM-MSCs). **d** Statistically significant compared to the diabetic group treated with anakinra (Anak)

**Fig. 10:**
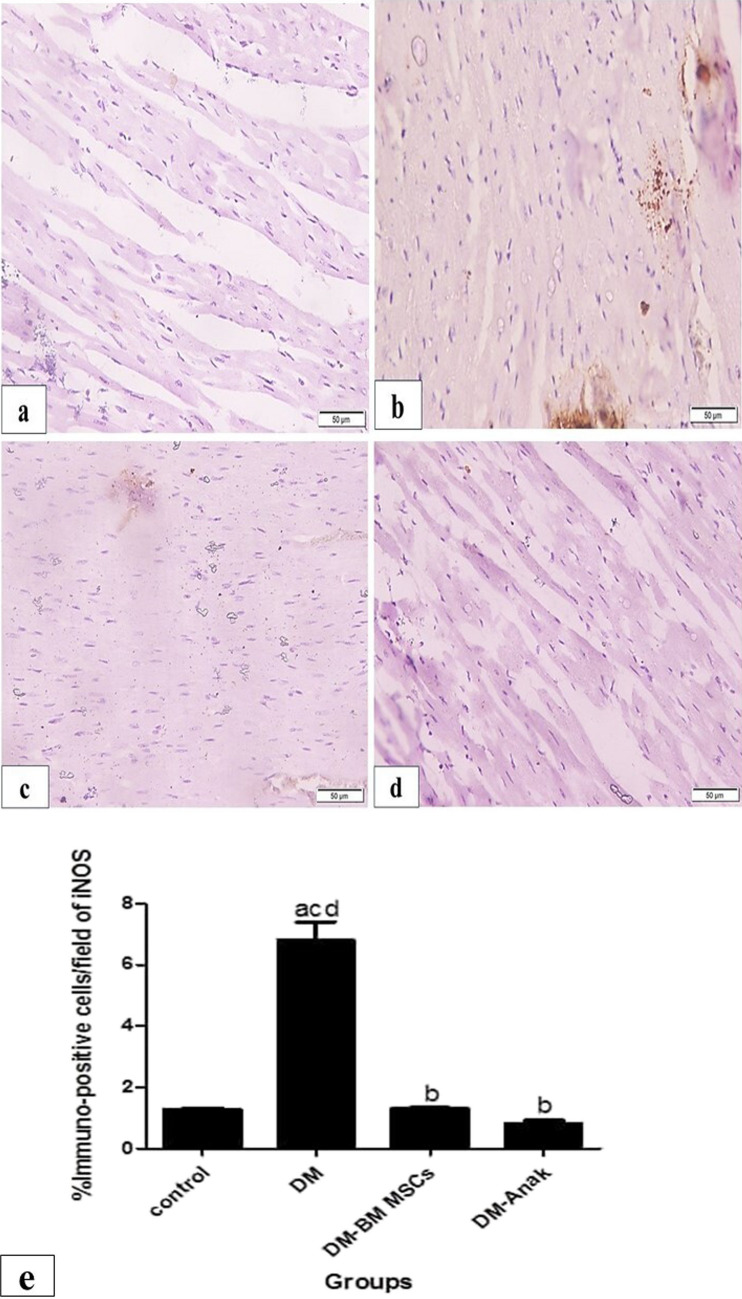
Immunoexpression of cardiac iNOS levels. **a** The control group showed minimal, mostly granular, and homogenous distribution, but in the diabetic non-treated group **b**, staining markedly increased, taking most of the fibers. The stem cell and Anak treated groups (**c**, **d**) showed less reaction, similar in pattern to the control group. **e** Percent area of iNOS; the DM group significantly increased immunostaining compared to control, while the treated groups significantly reduced its immunostaining compared to the DM group. Results of study were for eight observations compared as mean ± SD. Significance was if *p* < 0.05. **a** Statistically significant compared to the control group. **b** Statistically significant compared to diabetic untreated group (*p* < 0.05). **c** Statistically significant compared to the diabetic group treated with stem cell therapy (BM-MSCs). **d** Statistically significant compared to the diabetic group treated with anakinra (Anak)

Caspase1 and iNOS immunoexpressions were cytoplasmic. The reaction was minimal, mostly granular, and homogenous in distribution within cardiac fibers in control group. The DCM group showed a significant increase in staining that was mostly homogenous, taking most of the fibers. Interestingly, the treated groups had significantly lower immunostaining as compared to the DCM group (Figs. 9 and 10).

### Effect of BM-MSCs or Anak on serum inflammatory markers

Serum TNFα and IL10 were not significantly different at base line in all groups; DCM showed a significant elevation in TNFα and reduction in IL10; both markers were significantly improved with MSC or Anak (Table 3).

**Table 3:** Serum inflammatory markers in all groups

Groups	Control	DM	DM-MSC	DM-Anak
TNFα	**32.40 ± 2.06**	**220.4 ± 13.95acd**	**116 ± 13.23abd**	**60.75 ± 4.1abc**
IL10	**8.68 ± 1.38**	**2.5 ± 0.5acd**	**14.63 ± 3.21ab**	**15.87 ± 1.8ab**

Results of study were for eight observations compared as mean ± SD. Significance was if *p* < 0.05. a: Statistically significant compared to the control group. b: Statistically significant compared to the diabetic untreated group (*p* < 0.05). c: Statistically significant compared to the diabetic group treated with stem cell therapy (BM-MSCs). d: Statistically significant compared to the diabetic group treated with anakinra (Anak)

### Measurement of CD11b and CD8 tissue levels using western blotting

**Fig. 11:**
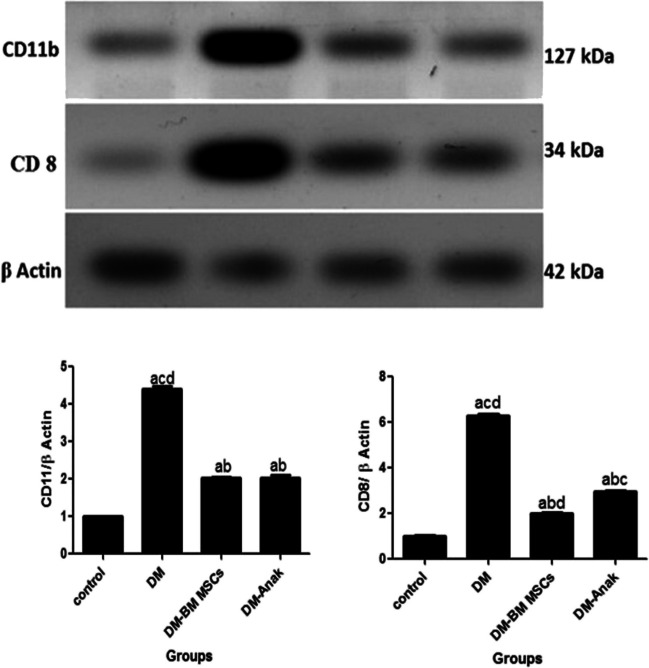
Evaluation of CD11b and CD8 using western blotting. By measuring protein level of CD11b and CD8 in all groups. The DM group showed significant increase compared to the control group, while the MSC or Anak therapy groups showed significant reduction in macrophage and T cell markers compared to diabetics. Anak administration significantly decreased CD 11b than BM-MSCs therapy. Results of study were for eight observations compared as mean ± SD. Significance was if *p* < 0.05. **a** Statistically significant compared to the control group. **b** Statistically significant compared to the diabetic untreated group (*p* < 0.05). **c** Statistically significant compared to the diabetic group treated with stem cell therapy (BM-MSCs). **d** Statistically significant compared to the diabetic group treated with anakinra (Anak)

CD11b and CD8 levels significantly increased in the DM group compared to the control group. However, the MSC or Anak given groups showed a significant decrease in macrophage and T cell markers compared to diabetics (Fig. 11).

## Discussion

One of the common consequences of long-standing DM-induced metabolic abnormalities is DCM (Grubić Rotkvić et al. 2021). Inadequate treatment of DCM can result in adverse cardiac remodeling and HF. It is well known that diabetic cardiac changes can be largely initiated by inflammation (Blasio et al. 2020). Therefore, in this study, we have used two approaches (BM-MSCs and Anak) to detect the effect of abrogating inflammatory pathways. BM-MSCs and Anak ameliorated cardiac structure and function together with reduced inflammatory markers and inflammation programmed cell death.

Indeed, utilizing the rat model of type II DM has been successfully used and faithfully simulated cardiac and structural changes of DCM. In the present study, rats received a HFD diet for 6 weeks to induce insulin resistance followed by a single STZ dose of 35 mg/kg while continuing the same HFD. Eight weeks later, this protocol resulted in significant deterioration in EF, FS, and enlarged diastolic dimensions. At the same time, signs of early diastolic dysfunction and reduced *E*/*A* ratio together with prolongation of deceleration time was observed. These cardiac changes remained persistent till the end of study and were associated with elevated fasting glucose, HOMA IR, and reduced serum insulin. When cardiac tissue samples were analyzed, fibrosis and collagen type III significantly increased. Assessment of IL1β, TNFα, and caspase1 in cardiac tissue revealed high levels, while Nrf 2 level was decreased. We have also found high cardiac tissue level of M1 macrophage marker CD11b, cytotoxic T cell marker CD8 together with high iNOS, most probably a hallmark marker of M1 macrophages (Xue et al. 2018). Grubić Rotkvić et al. (Grubić Rotkvić et al. 2021) and Chandramouli et al. (Chandramouli et al. 2018) reported diastolic and mild systolic dysfunction with fibrosis and enlarged LVEDD. On the contrary, functional causes such as disrupted calcium homeostasis can cause dilated cavities without evident fibrosis (Marchini et al. 2020).

There are numerous cross talks between metabolic and inflammatory signaling pathways in DM. Endogenous lipids activate toll like receptor 4 (TLR4) that mediate both inflammation and insulin resistance in adipose tissue with paralleled activation of NF-κB. Consequently, the diabetic environment comprises elevated proinflammatory cytokines such as TNFα, IL1β, and IL6 that could enhance NLRP3 inflammasomes, caspase 1, and maturation of IL1β. A positive feedback loop develops between TNFα, NF-κB, and IL1β, accentuating leukocyte formation with further activation of inflammation and apoptosis (Kaur et al. 2021). Supporting this, the predominance of M1 macrophages in DM and its role in promoting DCM has been previously reported (Lehrke et al. 2004; Wang et al. 2023; Coggins and Rosenzweig 2012).

The NLRP3 inflammasome is a key sensor mediating innate immune and inflammatory responses. It consists of NLRP3, apoptosis-associated speck-like protein containing a CARD domain (ASC) and pro-caspase1. ASC is the adaptor protein that links NLRP3 and pro-caspase1. Elevated proinflammatory cytokines as TNFα and IL1β in DCM and binding to their cognate receptors TLR-4–MyD88 and IL1 β receptor on cardiomyocytes cause activation of NF-κB (Kaur et al. 2021). Upon activation by NF-κB, NLRP3 is oligomerized and interacts with ASC which then interacts with pro-caspase1, triggering autocleavage of pro-caspase 1 to become active caspase1 which is involved in pyroptosis triggered fibrosis in DCM. Active caspase1 leads to cleavage and maturation of pro-IL 1β to become IL 1β. Interestingly, the important role of the NLRP3 inflammasome in DCM progression was discussed in different studies that examined the role of some pharmacological approaches in enhancing DCM via regulating the NLRP3 inflammasome. For example, administration of the anti-aging protein Klotho for 12 weeks in DCM mice suppressed thioredoxin interacting/inhibiting protein expression, NLRP3 inflammasome activation, and IL1β. Additionally, cardioprotective effect of rosuvastatin in DCM rats was abrogated with NLRP3-miRNA treatment confirming the essential role of NLRP3 inflammasome in DCM model (Sun and Ding 2021).

These DM mediated cardiac changes were reversed by systemic administration of BM-MSCs. The role of MSCs in tissue repair is related to its paracrine immunomodulation, thereby reducing myocardial inflammation, apoptosis, and fibrosis (Silva et al. 2022). We have also here detected that BM-MSCs reduced fibrosis and cardiac levels of IL1β, TNFα, concomitant to suppression of inflammasome stimulated caspase1, the activator of IL1β that is in accordance with previous studies (Sun and Ding 2021; Shams Eldeen et al. 2019; Franchi et al. 2009). BM-MSCs secrete IL-R antagonist (IL1-Ra) that acts in a paracrine/endocrine manner suppressing myocyte production of IL1β and limiting the feedback inflammatory loop of TNFα and inflammasome mediated caspase1 (Kaur et al. 2021; Shams Eldeen et al. 2019; Franchi et al. 2009; Volarevic et al. 2010; Chandramoorthy et al. 2018). In addition, BM-MSCs significantly reduced iNOS level, metabolic inflammation, oxidative stress, and macrophage M 1 activity.

The regulating role of BM-MSCs in M1/M2 polarization has been documented (Kuppa et al. 2022). In addition, recent studies have provided important mechanistic insights of BM-MSC biology in the vicinity of inflammation and its ability to decrease the intra cellular levels of IL1β, and TNFα as well as their levels in macrophages thus reducing M1 macrophages activation and consequently iNOS expression which have essential roles in mediating DCM (Jin et al. 2022). This priming effect has been demonstrated in healing of wounds, liver tissues, corneal epithelium, and nervous tissue, while not yet demonstrated in cardiac tissues (Redondo-Castro et al. 2017; Vander Beken et al. 2019; Harrell et al. 2020). Furthermore, preconditioning of BM-MSCs by IL1β selectively promotes their migration to various target organs and improved stem cell induced M2 polarization and M1 reduction (Saparov et al. 2016; Philipp et al. 2018; Jesmin et al. 2006; Wang et al. 2022).

In accordance, we have similarly observed a role for IL1β in primming stem cells against M1 macrophages in cardiac tissues and detected significant reduction of the M1 macrophage marker CD11b and cytotoxic T cell marker CD8 in the MSC treated group. In addition, it is found that BM-MSCs increased cardiac levels of the anti-inflammatory NrF-2 that decreases inflammation through interaction with NF-κB and direct inhibition of IL1β and IL18. Furthermore, NrF-2 reduces ROS levels through activating antioxidant target genes, inhibiting NLRP3 inflammasome priming (Helou et al. 2019) and consequently repressing pyroptosis and caspase1 activation. It is worth noting here that despite the promising role of stem cell therapy in DCM, its use is challenging regarding its survival in diabetic milieu, risk of immunological rejection, and its cost (Klerk and Hebrok 2021). The standardization, quality, and consistency of MSC-based therapy still require extensive future research (Silva et al. 2022).

In the present study, we have also explored the effect of IL1β receptor direct blocking using Anak. We detected improvement in cardiac echo parameters as well as serum glycemic and inflammatory profiles with lower cardiac caspase1, IL1β, and TNFα and reduced cardiac fibrosis and LVEDD. We also have observed a significant reduction in iNOS, CD11b, and CD8 levels. In addition, mitral inflow velocities were also significantly enhanced. This is due to Anak ability to block IL1R that is responsible for activation of the mediators of hypertrophy, fibrosis, and cardiac contractile dysfunction and its ability to induce oxidative stress and inflammatory immune responses (Pollack et al. 2016; Volarevic et al. 2010). This is in line with Amin et al. (Amin et al. 2012) who reported that Anak significantly enhanced ischemia-induced neovascularization, reduced ER stress markers, and reduced macrophage infiltration in the ischemic hind-limb of mice. Our results are also in accordance with different previous models (Vallejo et al. 2014; Lacraz et al. 2009; Ehses et al. 2009).

More recently, Vallejo et al. (Vallejo et al. 2014) indicated improved endothelial dysfunction in T2DM rat aortic and mesenteric vessels following Anak administration that was associated with reduced serum levels of IL1β and TNFα. In addition, previous study using Anak in an effective dose of 1 mg/kg/day or 100 mg/kg/day for 1 or 2 weeks ameliorated cardiac echo parameters and reduced cardiac apoptotic markers in a model of acute myocardial infarction (Salloum et al. 2009). However, all of these studies did not explore a direct effect of Anak on DCM. This is the first study to report Anak induced IL1β receptor blocking to improve DCM function.

Anak has a good safety profile as an anti-inflammatory drug traditionally used in scenarios with features of hyperinflammation and recently found to be safe in improving inflammation in COVID-19 patients (Fanlo Mateo et al. 2023). However, using Anak may be challenging regarding its cost, availability, and short half-life. Risk of infection during treatment with Anak needs to be taken seriously only during high-dose therapy in patients with significant co-morbidities. In CANTOS study, canakinumab (monoclonal antibody against IL 1β) showed no improvement in glycemic control in diabetic patients while Anak achieved better glycemic control by improving HbA1C and proinsulin to insulin ratio (Mikkelsen Rasmus et al. 2022). Additionally, canakinumab failed to reduce the risk of cardiovascular events in type 2 DM (Tan et al. 2020).

In conclusion, Anak and BM-MSC therapy are two novel therapeutic approaches that clearly improved the inflammatory responses in DCM by suppressing TNFα/ILlβ/caspase 1, macrophage, and CD8 T-cell.

## Conclusion

IL1β receptor antagonism (Anak) and BM-MSC therapy are effective in mitigating myocardial inflammation and improving cardiac performance in DCM. Further studies regarding administration of Anak and BM-MSC therapy to patients of DCM should be considered and in critical need.

## Data Availability

Data is available upon request. All authors read and approved the submission of the manuscript.

## Abbreviations

*A* wave: Late diastolic filling velocity
Anak: Anakinra
BM-MSC: Bone marrow mesenchymal stem cell
DCM: Diabetic cardiomyopathy
DM: Diabetes mellitus
DT: Deceleration time
EF: Ejection fraction
*E* wave: Peak early diastolic filling velocity
FBG: Fasting blood glucose level
FS: Fractional shortening
HFD: High-fat diet
IL1β: Interleukin 1β
IL10: Interleukin 10
iNOS: Inducible nitric oxide synthase
LVEDD: Left ventricular end diastolic diameter
LVESD: Left ventricular end systolic diameter
NF-κB: Nuclear factor kappa-B
NLRP3: NLR family pyrin domain containing 3
